# Epigenetic Targeting of Glioblastoma

**DOI:** 10.3389/fonc.2018.00448

**Published:** 2018-10-16

**Authors:** Massimo Romani, Maria Pia Pistillo, Barbara Banelli

**Affiliations:** ^1^Laboratory of Tumor Epigenetics, IRCCS Ospedale Policlinico San Martino, Genova, Italy; ^2^Department of Health Sciences, University of Genoa, Genova, Italy

**Keywords:** glioblastoma, epigenetics, therapy, DNA methylation, histone code

## Abstract

Glioblastoma is one of the first tumors where the biological changes accompanying a single epigenetic modification, the methylation of the *MGMT* gene, were found to be of clinical relevance. The exploration of the epigenomic landscape of glioblastoma has allowed to identify patients carrying a diffuse hypermethylation at gene promoters and with better outcome. Epigenetic and genetic data have led to the definition of major subgroups of glioma and were the basis of the current WHO classification of CNS tumors and of a novel classification based solely on DNA methylation data that shows a remarkable diagnostic precision.The reversibility of epigenetic modifications is considered a therapeutic opportunity in many tumors also because these alterations have been mechanistically linked to the biological characteristics of glioblastoma. Several alterations like *IDH1/2* mutations that interfere with “epigenetic modifier” enzymes, the mutations of the histone 3 variants H3.1 and H3.3 that alter the global H3K27me3 levels and the altered expression of histone methyltransferases and demethylases are considered potentially druggable targets in glioma and molecules targeting these alterations are being tested in preclinical and clinical trials. The recent advances on the knowledge of the players of the “epigenetic orchestra” and of their mutual interactions are indicating new paths that may eventually open new therapeutic options for this invariably lethal cancer.

## Introduction

Glioblastoma (Glioblastoma Multiforme, GBM) is a rare tumor (Orphanet 360) that, being responsible for 4% of all tumor deaths and with a 5-years survival of 2%, is one of the deadliest human tumors (1) with the median survival ranging from 14 to 24–30 months depending from the molecular subtype of the tumor (2).

GBM, like other tumors, harbors many genetic alterations that interfere with cancer-related pathways (3), however clinical trials targeting molecular alterations in this tumor were largely unsuccessful so far (4–6). In the last 30 years, the only significant improvement in OS occurred with the introduction of Temozolomide (TMZ) in addition to surgery and radiotherapy (7, 8). GBM patients are stratified into two categories according to the methylation status of the O-6-methylguanine-DNA methyltransferase gene (*MGMT)* that repairs the DNA damages induced by TMZ and the patients whose tumor contains methylated *MGMT* have an overall survival of 21.7 months compared to the 12.7 months of those carrying unmethylated *MGMT* (9).

**Figure 1:**
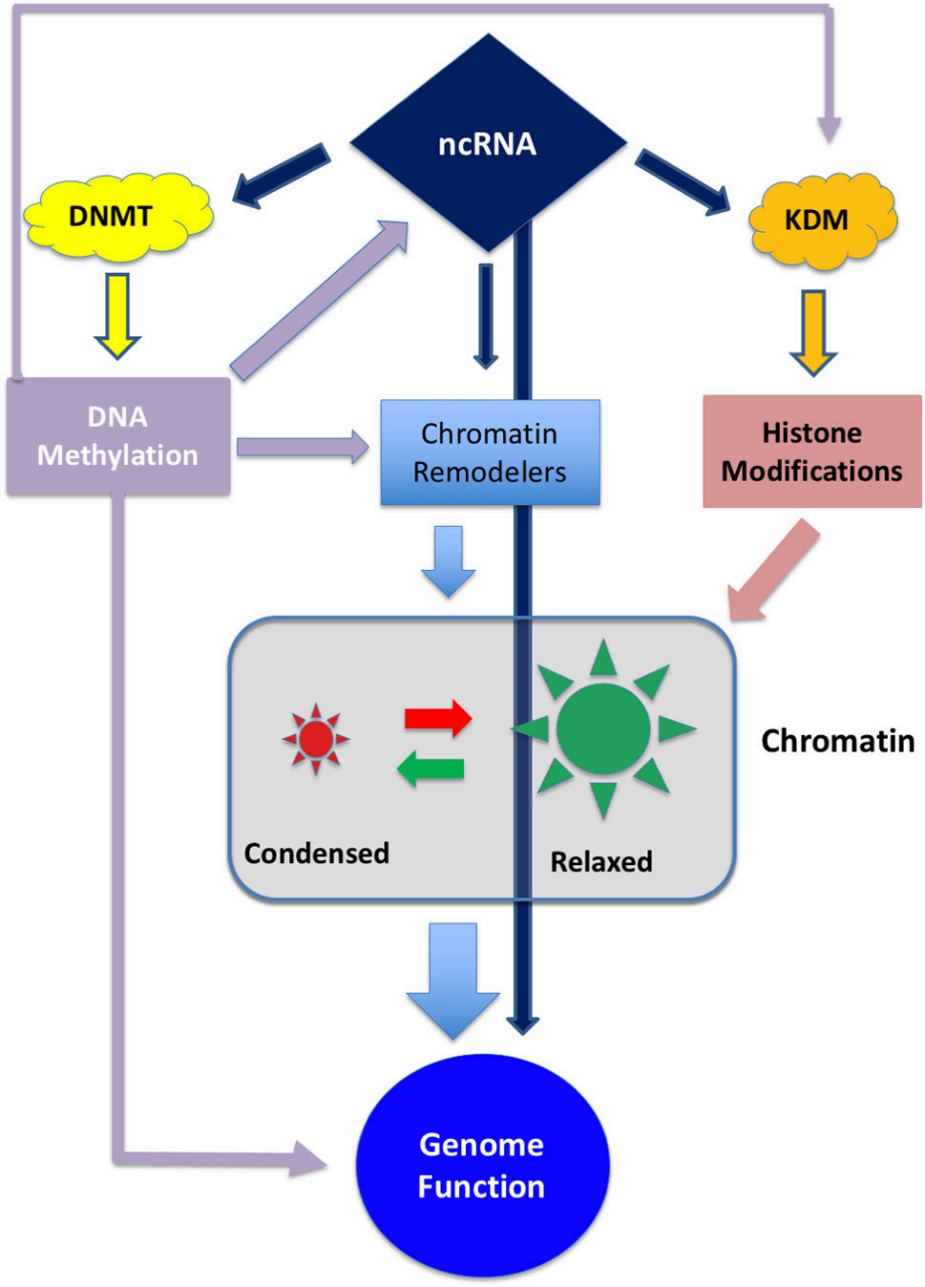
Schematic representation of the interplay between the different epigenetic layers. ncRNA can directly influence genome activity by interfering with transcripts or, indirectly, by degrading transcripts involved in DNA methylation, histone modification or chromatin remodeling. On the other hand ncRNA can be epigenetically inactivated by DNA methylation (11, 12). DNA methylation can directly interfere with gene expression and, indirectly, can regulate the expression of chromatin and histone modifiers.

Epigenetic modifications are considered a key mechanism in GBM development (10). Epigenetic inheritance is mediated by the four deeply interconnected layers shown in Figure 1:

1- DNA methylation
2- Histone modifications
3- Chromatin remodeling
4- Non-coding RNA

These layers are controlled by a set of enzymes that act as “writers,” “readers,” and “erasers” that modify their target by adding, removing or regulating the interactions between proteins and DNA. Both DNA methylation and histone modification, along with chaperon molecules, participate to chromatin remodeling thus conferring an exquisite plasticity to the genetic apparatus (13, 14).

**Figure 2:**
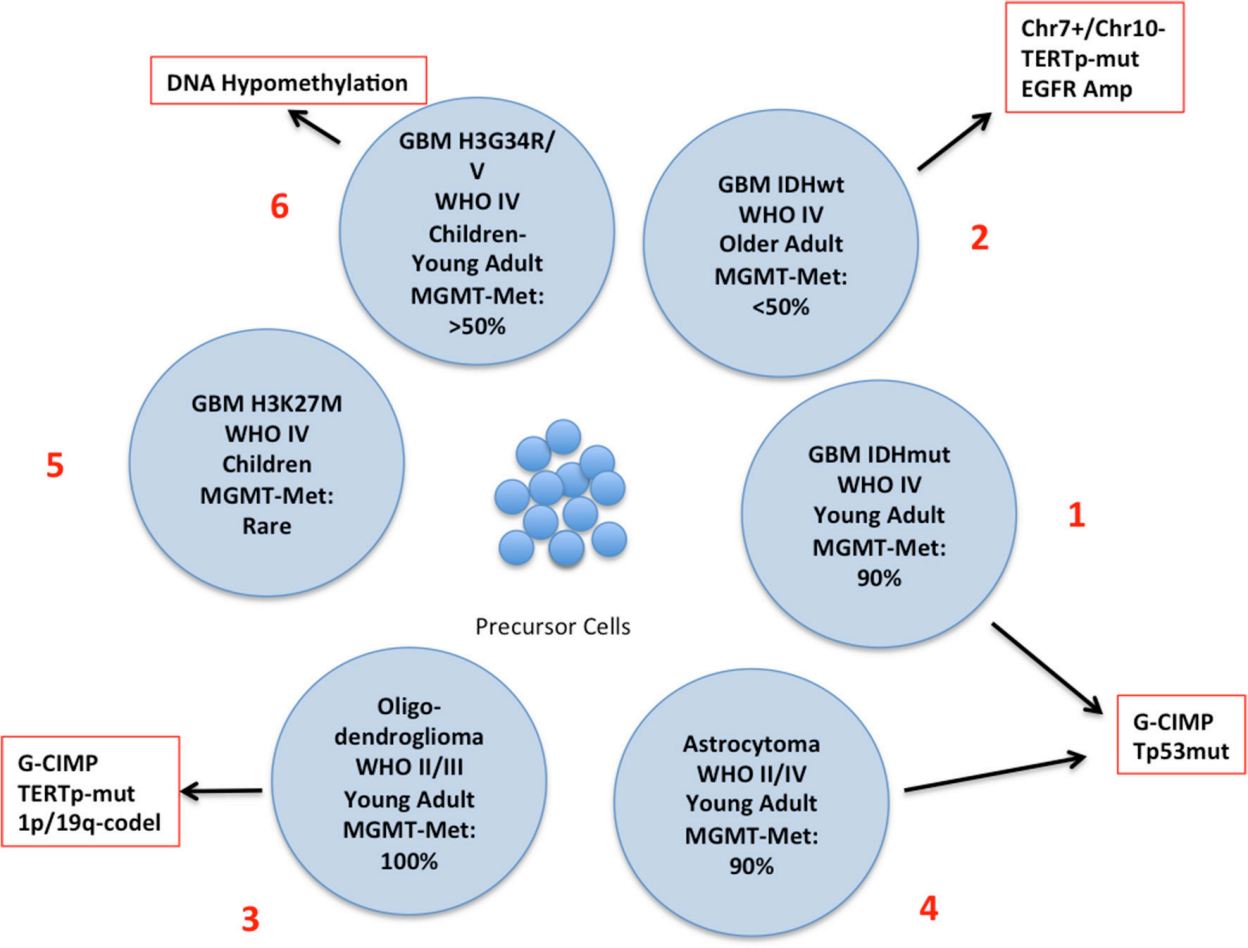
Schematic representation of the different subtypes of glioma with the principal molecular and epigenetic characteristics. In this chart are represented the possible carcinogenic evolutions of the precursor cells. The major genetic and epigenetic alterations are reported along with the clinical characteristics of each subtype.

The latest WHO classification defines subgroups of glioma integrating genetic and epigenetic criteria (10, 15–18) (Figure 2) and a novel classification of CNS tumors based on DNA methylation data, shows a remarkable diagnostic precision being able to correctly modify the primary diagnosis in 12% of the cases (19). A large number of intrinsically reversible cancer-related modifications that are attractive targets of therapy was unveiled and the present review provides an overview of the most recent preclinical and clinical attempts to defy GBM through epigenetic reprogramming.

## Targeting epigenetic alterations in glioblastoma

Manipulating the epigenome has been lengthy considered a therapeutic opportunity in cancer. The epigenetic landscape of GBM was thoroughly explored, and many epigenetic modifications were mechanistically linked to the biological characteristics of this tumor and some of them were considered as therapeutic targets. At the moment, only the molecules acting on DNA methylation and histone methylation/chromatin remodeling were tested in clinical trials. Manipulation of ncRNA expression is restricted to pre-clinical studies and is not discussed in the present review.

### Layer 1: DNA methylation

Methylation of cytosine at C-5 within CpG doublets, is mediated by a set of DNA Methyltransferases that are responsible mainly but not exclusively of maintenance (DNMT1) and *de novo* DNA methylation (DNMT3a and 3b) to preserve genomic integrity (20). The human genome contains approximately 3 X 10^7^ CpG doublets and although methylation at single doublets may, in principle, have functional consequences (21, 22), the biologically-relevant DNA methylation is that occurring at CpG clusters (CpG islands) in gene promoter regions and inversely correlates with gene transcription (23, 24). Intragenic CpG clusters are generally hypermethylated to prevent spurious initiation particularly at internal promoters.

In GBM DNA methylation is tightly linked to the response to TMZ treatment. The alkylating agent TMZ, the first-line chemotherapy for GB, methylates guanine in position N^7^ and O^6^ and Adenine in position N^3^. O^6^-methylguanine adducts lead to strand breaks, triggering p53-mediated apoptosis through the Fas/CD95/Apo-1 receptor in p53wt cells or through the mitochondrial pathway in p53mut tumors (25). The action of TMZ is counteracted by the MMR system and by the product of the *MGMT* gene that repairs the O^6^ adducts that limit the activity of the drug (26). To mimic the effects of *MGMT* methylation, synthetic inhibitors of MGMT entered human trials (9, 27). However, several studies revealed that the MGMT inhibitors O^6^-benzylguanine and PaTrim-2 (Lomeguatrib) did not improve the response rate to TMZ and increased the adverse effects of chemotherapy (27–30).

Inducing TMZ sensitivity in *MGMT*-unmethylated tumors with other molecules (Resvetrol, oncolytic viruses or by MGMT depletion) was tested only in preclinical models with alternate success (31). The correlation between *MGMT* methylation and MGMT protein expression is controversial and the lack of correlation seen in recent studies likely depends on the early method utilized for methylation analysis (32–34).

### Layer 1: methylator phenotype and *IDH1/2* mutations

The discovery of mutations of the Isocitrate Dehydrogenase (*IDH*) genes and of the DNA hypermethylation signature (*G*lioma *C*pG *Island Methylator* Phenotype: G-CIMP) has led to the definition of a distinct GBM subtype characterized by younger age and improved survival (2, 17, 18, 35) (Figure 2, N. 1). *IDH* mutations are rare in GBM developing in older patients who usually carry EGFR and PTEN alterations (primary GBM), (Figure 2, N. 2), but are present in a large proportion of low-grade glioma and, along with TP53 mutations, in high-grade glioma that evolved from low–grade tumors (secondary glioblastoma) (35) Figure 2, N. 3 and 4). IDH genes can be mutated at two mutually exclusive sites, R132 (*IDH1*) or R172 (*IDH2*) and these mutations have important metabolic consequences and are driving alterations in gliomagenesis. The product of *IDH* converts Isocitrate into αKetoglutarate (αKG) which is involved in a variety of cellular processes (Supplementary Figure 1). IDH mutants produce 2-Hydroxy Glutarate [2-HG] that is a competitive inhibitor of αKG-dependent dioxygenases including the histone demethylases JHDM1 and KDM4 and the DNA demethylase TET2. Thus, IDH mutations, that are not restricted to brain tumors, result in extensive epigenetic dysregulation including DNA and histone hypermethylation (36, 37) and altered cell differentiation (38). Other IDH mutations were occasionally found but only few of them produce 2-HG (39).

Strategies to target IDH-mutant tumors can be designed to either inactivate the functions of IDH mutants or to block the effects of 2-HG. The treatment with hypomethylating agents of mice xenografted with IDHmut GBM cells resulted in delayed tumor growth and improved survival (40, 41). Along this line phase I and II clinical trials were started to test two formulations of 5-azacytidine (NCT02223052) and the combination decitabine/immunotherapy (NCT02332889) in GBM and other solid tumors.

Normalizing the 2-HG concentration could reverse DNA hypermethylation and release the block of differentiation in IDH-mutated cells. Several inhibitors of mutated IDH1/2 were synthesized and showed to be effective in *in vitro* models (42–44); this finding was the starting point for a large series of clinical trials to assess the safety and bioavailability of the molecules under investigation in a variety of tumors, mainly AML, MDS and glioma (Supplementary Table I). Preliminary data on the clinical efficacy of IDH inhibitors showed promising results in hematological malignancies opening the way for stringent randomized trials (45–47). As of June 2018, no public data are yet available for glioma patients.

Mutated IDH1/2 can be functionally considered as highly specific tumor-associated neoantigens that could be targeted by immunotherapy; a vaccine targeting mutant IDH1 showed antitumor activity in a glioma animal model opening the possibility of new experimental therapies (48).

### Layer 2: histone modifications

Histones are subject to modifications that could either repress or activate transcription (Supplementary Figure 2). More than 100 enzymes act in concert to assemble a “code” of histone modifications that define the transcriptional properties of a given gene (49) determining drug response (50) and the development of cancer and other diseases (10, 51–53).

The rapid acquisition of drug resistance is a major cause of treatment failure in GBM (54) and could be explained by the development of epigenetically poised cells that undergo chromatin remodeling and display transient drug resistance (55–57).

### Histone acetylation

The addition of acetyl groups to certain lysines of H3 and H4 weakens the interaction between the core histones and DNA favoring the accessibility of the transcription apparatus. Deacetylation removes the acetyl groups provoking chromatin condensation and gene inactivation (49, 58). Acetylation and deacetylation are dynamic processes mediated by histone acetyltransferases (HAT) and histone deacetylases (HDAC) that maintain the balanced state of acetylation. Gain of HDAC expression has been found in many tumors, including GBM, and inhibitors of HDAC (HDACi) have been extensively explored for GBM therapy. HDACi have a large spectrum of antitumor activity and six HDACi have been approved by FDA: Vorinostat (11 studies concluded and 3 ongoing), Romidepsin (one study concluded), Belinostat (one study ongoing), Panobinostat (2 studies terminated before completion), Valproic acid (two studies terminated before completion and two recruiting) and Entimostat (no studies yet) (59) (https://clinicaltrials.gov, June 2018). Preclinical studies have shown that HDACi are very effective against GB cells, but the results of the clinical trials were largely disappointing. In adult patients Vorinostat was utilized as single agent and in combination with standard or biological therapies and in one study (NCT00238303) prolonged disease stabilization in a small subset of patients when used as single agent (60) but its addition to the standard radio/chemotherapy did not improve survival (61). Phase I/II trials with Romidepsin, with Panobinostat and anti-VEGF, or with Vorinostat and the proteasome inhibitor Bortezomib were either ineffective or toxic and were discontinued (62–64). Panobinostat however, is now being tested as a radiosensitizing molecule with promising results (65). Along this line phase II studies demonstrated that the addition of valproic acid to the standard radio/chemotherapy or to radiotherapy alone improved survival (66, 67). Randomized trials are necessary to confirm this finding.

### Histone methylation

Histone methylation was discovered along with histone acetylation (58) but its function remained obscure for many years because methylation does not change the DNA/protein interactions and it seemed an irreversible modification. With the discovery of LSD1 (KDM1), the first histone demethylase, it became clear that histone methylation is a reversible process (68) mediated by approximately 30 enzymes subdivided into distinct classes, and linked to a variety of physiological and pathological conditions including cancer, cardiovascular diseases, abnormal immune response and neurological disorders (51, 69). Histone methylation involves certain lysine and arginine of H3 and H4 and can either activate or repress transcription (Supplementary Figure 2).

In glioblastoma, histone methylation has distinct implications in pediatric and adult patients. Histone variant H3.3 (*H3F3A*) marks active chromatin domains and in pediatric tumors can be mutated at two sites: lysine 27 (K27M) and glycine 34 (G34R/V) (70, 71) (Figure 2, N. 5 and 6). In pediatric glioma the K27M mutation is restricted mostly to midline tumors whereas G34R/V is prevalent in hemispheric gliomas. K27M decreases methylation at K27 leading to transcriptional activation. G34R/V is associated with the redistribution of the activation mark H3K36 methylation and results in the upregulation of the oncogene *MYCN* (72) whose exogenous overexpression initiates glioma formation during development (73). *H3F3A*-K27M also inhibits the PRC2-EZH2 axis (2, 74), that acts as a histone methyltransferase, leading to the generalized loss of H3K27 methylation and to the CpG hypomethylator phenotype (CHOP) whose consequence is the aberrant activation of gene expression (75).

The methylation of H3K27 is regulated by PRC2-EZH2 methylases and by the UTX (KDM6A) and KDM6B demethylases; the effect of the K27M mutation could be reversed by inhibiting H3K27 demethylation. In an experimental model of diffuse intrinsic pontine glioma (DPIG), the small molecule GSK J4 was utilized to inhibit the activity of KDM6B (76). It was found that GSK J4 passes the Blood Brain Barrier and prolongs survival of mice xenografted with H3K27 tumors but not that of mice carrying WT H3.3 or G34R/V tumors. Although GSK J4 has proven to be effective in *in vivo* tumor models as single agent or synergically with HDACi (76–78), as of June 2018, clinical trials employing this or similar molecules have not been launched yet.

Targeting EZH2 is another mechanism to modulate histone methylation and to reverse tumor growth (79). Several FDA-approved EZH2 inhibitors are available (Tazemetostat, CPI-1205, GSK2816126) and others are in advanced pre-clinical testing. More than 20 trials that include EZH2 inhibition are reported in the Clinical Trial database mostly aimed at hematological disorders. As of June 2018, most studies are still ongoing and recruiting. However, studies with Tazemetostat (NCT03213665; NCT03217253) were suspended because of adverse events and one study with GSK2816126 (NCT02082977), was interrupted because of insufficient evidences of clinical response.

Mutations of H3.3-*H3FA* are uncommon in adult GBM where H3.3 can be functionally inactivated by the *MLL5* gene that is overexpressed in GBM stem cultures (80). Finally, it was found that GSK J4, like in pediatric GBM, has strong suppressive effects on cell viability and self-renewal properties (80, 81).

Several histone demethylases are constitutively or transiently overexpressed in adult GBM. LSD1 (KDM1) is FAD-monoamine oxidase that demethylates several lysine of H3 (K4, K9, K27, and K36). KDM1 interacts with non-histone substrates and inhibits p53 activity by demethylating K370me1 and by inhibiting the interaction with the coactivator 53BP1 (82). Inhibitors of KDM1 derive mainly from MAO inhibitors utilized in the clinical practice and are strong suppressors of tumor cell proliferation *in vitro* and in animal models (83). Most KDMi are non-selective for KDM1 and have additional irreversible activity on MAO. As of June 2018, three molecules were approved by FDA for clinical utilization (GSK2879552, IMG-7289 and INCB059872) in addition to the antidepressants Tranylcypromine and Phenelzine whose antitumor activity is being explored in phase I trials. Some of these trials were prematurely concluded because of toxicity and low efficacy while others are still ongoing.

In GBM, recurrence occurs from residual cells at the margin of resection that rapidly acquire radio- and chemo-resistance during treatment and cannot be efficiently counteracted by other drugs.

The induction of drug resistance is accompanied by the overexpression of several *KDM* genes. Indeed it was shown that upon treatment, a restricted population of slow-cycling cells undergo epigenetic, thus reversible, changes that result in drug resistance and sustained tumor growth (55, 56). A key effector of this mechanism is the H3K4 demethylase KDM5A gene whose exogenous expression or inactivation mimics drug resistance and sensitivity in different tumors including GBM (55, 56, 84, 85). Overall many pre-clinical and clinical evidences indicate that the entire KDM5 family, as well as other KDMs are emerging targets in cancer therapy (69, 86–89).

The pan-KDM inhibitor JIB 04 is maximally active against KDM5A but, at lower efficacy, inactivates also other KDMs found overexpressed in TMZ-resistant GBM cells (85) and has a strong antitumor effect (90). JIB 04 was utilized in a model of acquired TMZ resistance and shown to ablate TMZ-resistant cells, to synergize with TMZ at clinically-relevant concentrations and finally, in a pilot experiment, to have promising activity *in vivo* (91). Similar effects were obtained with CPI-455, a selective inhibitor of KDM5 (92) but at a concentration difficult to reach *in vivo* (91, 92). Similarly, NSCLC cells that acquired resistance to taxane/platinum combinations became sensitive to JIB 04 and GSK J4 that reverted, at least in part, the transcriptional program of resistant cells to that of drug-naïve cells and synergize with standard chemotherapy as JIB 04 and Temozolomide (93). As of June 2018, none of these promising molecules is being tested in clinical trials.

### Layer 3: chromatin remodeling

Changing chromatin conformation regulates accessibility to transcription factors, to the DNA replication and repair machineries. The proper chromatin conformation is determined by histones and their modifications and by the chromatin remodeling complexes that include the histone modifiers described in section Layer 2: Histone Modifications and the ATP-dependent chromatin remodeling complexes (SWI/SNF; ISWI; CHD and INO80) (94–96). These complexes include many components that play essential and redundant roles in normal cells and that are variably altered in most human cancers (97). Because of their complexity, chromatin remodelers are very difficult targets for drug discovery and the identification of their synthetic inhibitors is still in its infancy (94). The tumor suppressor SWI/SNF complex was the first chromatin remodeler discovered, is mutated in more than 20% of the tumors (97, 98) and is involved in the maintenance of stemness in glioma cells (99). The effects of SWI/SNF inactivation can be counteracted by inhibitors of the TK pathway and of NF-kB (100, 101); however, as outlined previously, these targeted therapies were unsuccessful in GBM patients. PARP-1 polymerase is involved in chromatin remodeling mechanisms through histone modification and inhibition of the ISWI complex (102). Two PARP inhibitors (Oliparib and Veliparib) were recently licensed by the FDA for ovarian cancer treatment and several other experimental molecules are undergoing extensive testing in humans and in animal models (103). For GBM, the NIH Clinical Trials Database reports seven ongoing or completed trials with Olaparib (104, 105) (NCT01390571, NCT03212274, NCT02974621) Veliparib (NCT02152982, NCT03581292, NCT01514201) and with BSI-201 (NCT00687765). The results of these studies were not yet disclosed.

Targeting of histone chaperon molecules in glioblastoma is just beginning to be explored, however promising results in animal models were obtained by targeting FACT, a nucleosome reorganization protein (106) with CBL0137 in combination with TMZ (107).

## Conclusions

Despite all the progresses in medicine, the median survival of GBM patients has not substantially improved, likely because this tumor rapidly becomes radio- and chemo-resistant and infiltrates the surrounding brain tissue making impossible the complete surgical eradication. To overcome this deadlock many experimental therapies were devised but none of them met the expected results. Epigenetic modifications are gaining strong relevance in glioblastoma because they can be either clinical biomarkers for the optimal stratification and classification of the patients and because they can be also potential drug targets as suggested by many preclinical trials. Molecules with epigenetic effects can potentially modulate the plasticity of the tumor environment in glioma and may drive the changes of the epigenomic environment restoring or rendering more susceptible the tumor cells to standard chemotherapy rather than be used as a monotherapy. In this respect the timing and the scheduling of the epigenetics and cytotoxic drugs could be crucial for the best clinical result and should be carefully defined on the basis of the chemical, biological and cellular effect of these treatments (91). Certainly, the addition of proteomic and metabolomic approaches to the extensive epigenomic and transcriptomic studies already conducted will have the capacity to unveil the inner mechanisms of glioma biology allowing the design of more effective drugs.

## Author contributions

MR conceived the idea of this mini-review article and wrote the first draft. BB, MP, and MR equally participated to the final writing of the article.

### Conflict of interest statement

The authors declare that the research was conducted in the absence of any commercial or financial relationships that could be construed as a potential conflict of interest. The reviewer XC and the handling editor declared their shared affiliation at the time of the review.

## Supplementary material

Supplementary Figure 1 IDH1/2 pathways. Metabolic pathways involving IDH1 (cytoplasmic) and IDH2 (mitochondrial). IDH1/2 (wt) converts Isocitrate into αKetoglutarate (αKG) while the mutated forms convert Isocitrate into 2-hydroxyglutarate that competitively inhibits αKG-dependent dioxygenases including the histone demethylases JHDM1 and KDM4 and the DNA demethylase TET2

Supplementary Figure 2 Schematic representation of the major H3 and H4 modifications and their functional role. In green and red the activating and the repressive modifications, respectively.

Supplementary Table 1 Clinical trials employing FDA-approved IDH1/2 inhibitors.
