# Supplementary figures and images for: Epigenetic Targeting of Glioblastoma
Source: Front Oncol. 2018 Oct 16;8:448. doi: 10.3389/fonc.2018.00448 (PMC6198064; doi:10.3389/fonc.2018.00448)

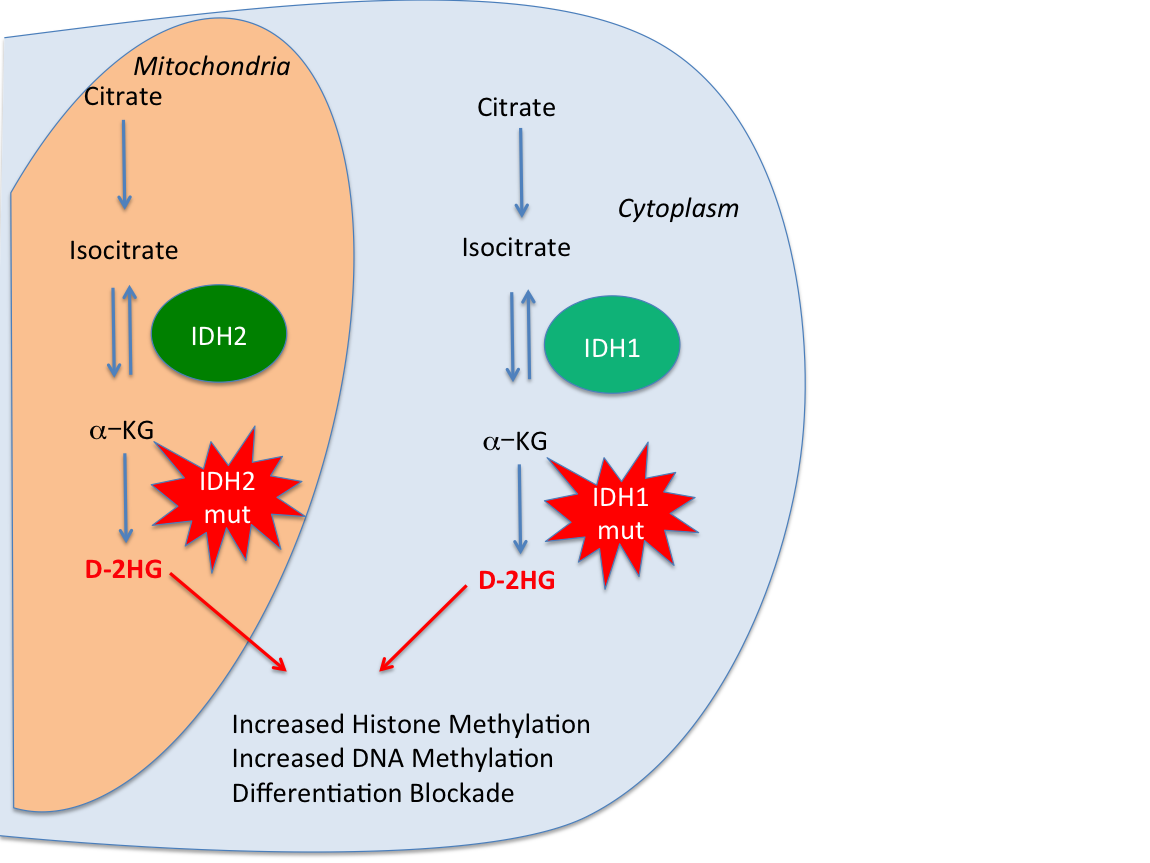

Supplement: Supplementary Figure 1 — IDH1/2 pathways. Metabolic pathways involving IDH1 (cytoplasmic) and IDH2 (mitochondrial). IDH1/2 (wt) converts Isocitrate into αKetoglutarate (αKG) while the mutated forms convert Isocitrate into 2-hydroxyglutarate that competitively inhibits αKG-dependent dioxygenases including the histone demethylases JHDM1 and KDM4 and the DNA demethylase TET2 [file Image_1.TIFF]

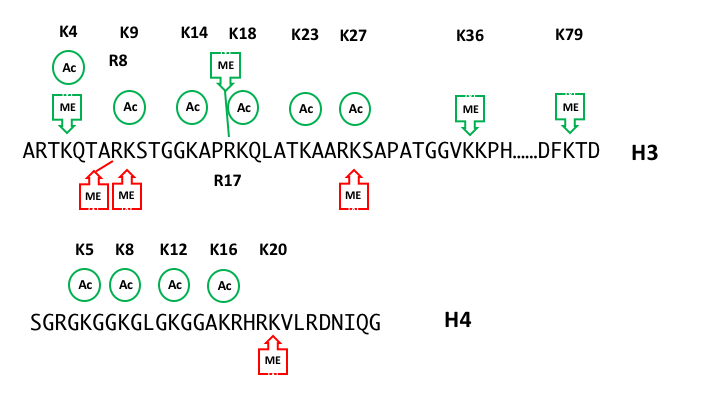

Supplement: Supplementary Figure 2 — Schematic representation of the major H3 and H4 modifications and their functional role. In green and red the activating and the repressive modifications, respectively. [file Image_2.TIFF]
