# Supplementary material for: Epigenetic Targeting of Glioblastoma
Source: Front Oncol. 2018 Oct 16;8:448. doi: 10.3389/fonc.2018.00448 (PMC6198064; doi:10.3389/fonc.2018.00448)
Supplement: Supplementary Table 1 — Clinical trials employing FDA-approved IDH1/2 inhibitors. [file Table_1.DOCX]

**Table 1** – Clinical trials employing FDA-approved IDH1/2 inhibitors

| Molecule | Clin. Trial ID | Tumor | Subjects Enrolled | Status | Notes |
| --- | --- | --- | --- | --- | --- |
| AG-120 | NCT033431 | Glioma | exp. 33 subjects | R | Phase I |
| AG-120 | NCT03071770 | Healthy subjects | 60 | C | Phase I |
| AG-120 | NCT02073994 | Solid Tumors | exp 170 subjects | NR | Phase I |
| AG-120 | NCT02074839 | AML | 266 subjects | AC | Phase I |
| AG-120 | NCT03503409 | MDS, AML | exp 66 subjects | NR | Phase II |
| AG-120 | NCT02989857 | Cholangiocarcinoma | exp. 186 subjects | NR | Phase III |
| AG-120 | NCT03173248 | AML | exp. 392 subjects | R | Phase III |
| AG-120 | NCT02489513 | Healthy subjects | 8 | C | Phase I |
| AG-120 | NCT02579707 | 36 Healthy subjects | 36 | C | Phase I |
| AG-120 | NCT02831972 | Healthy subjects | 22 | C | Phase I |
| AG-120 | NCT03471260 | Hematologic malignancies | exp. 48 subjects | R | Phase I/II |
| AG-120 | NCT03245424 | Pediatric and adult AML | NI | R | NI |
| AG-120 | NCT03564821 | AML | exp 22 subjects | NR | Phase I |
| AG-221 | NCT01915498 | Hematologic malignancies | Exp 357 subjects | NR | Phase I/II |
| AG-120 and AG-221 | NCT02632708 | AML | exp 144 subjects | R | Phase I |
| AG-120 and AG-221 | NCT02677922 | AML | exp 127 subjects | R | Phase I/II |
| AG-221 | NCT02218346 | Healthy subjects | 30 | C | Phase I |
| AG-221 | NCT02273739 | Healthy subjects | 22 | C | Phase I |
| AG-221 | NCT02443168 | Healthy subjects | 14 | C | Phase I |
| AG-221 | NCT02387866 | Healthy subjects | 60 | C | Phase I |
| AG-221 | NCT02577406 | AML | Exp 316 subjects | R | Phase III |
| Molecule | Clin. Trial ID | Tumor | Subjects Enrolled | Status | Notes |
| AG-221 | NCT03383575 | MDS | Exp 105 subjects | R | Phase II |
| AG-881 and AG-120 | NCT03343197 | Glioma | Exp 45 subjects | R | Phase 1 |
| AG-881 | NCT02492737 | MDS, AML | Exp 46 subjects | NR | Phase 1 |
| AG-881 | NCT02481154 | MDS, AML | Exp 150 subjects | NR | Phase I |
| IDH305 | NCT02987010 | Low grade gliomas | 0 subjects | WT (safety issues) | Phase I |
| IDH305 | NCT02977689 | Glioma | 0 subjects | WT (safety issues) | Phase II |
| IDH305 | NCT02381886 | Advanced Malignancies | Exp. 166 subjects | NR | Phase I |
| IDH305 | NCT02826642 | AML | 0 subjects | WT (safety issues) | Phase I |
| BAY1436032 | NCT02746081 | Advanced solid tumors | Exp 100 subjects | R | Phase I |
| FT-2102 | NCT02719574 | AML, MDS | Exp. 400 subjects | R | Phase I, II |

Data source: <https://www.clinicaltrials.gov> (June 2018)

R: Recruiting

C: Completed

NR: Not Recruiting

AC: Accrual completed

NI: Not Indicated

WT: Withdrawn
